# Supplementary material for: Transcriptome Analysis Revealed Hormone Pathways and bZIP Genes Responsive to Decapitation in Sunflower
Source: Genes (Basel). 2022 Sep 27;13(10):1737. doi: 10.3390/genes13101737 (PMC9601282; doi:10.3390/genes13101737)
Supplement: Supplementary file 1 [file genes-13-01737-s001.zip › genes-1890563-supplementary.pdf]

Table S1: Protein information of HabZIP TF.

Sequence ID	Number of Amino Acid	Molecular Weight	Theoretical pI	Instability Index	Aliphatic Index	Grand Average of Hydropathicity
HabZIP50	273	30059.57	5.81	32.06	61.1	-0.781
HabZIP47	252	27726	6.75	39.65	61.59	-0.728
HabZIP22	105	12410.67	11.3	68.34	83.62	-0.918
HabZIP40	221	24713.95	6.87	64.41	75.38	-0.739
HabZIP17	229	25210.96	9.93	60.16	56.72	-0.913
HabZIP49	234	25908.96	9.6	55.24	69.62	-0.725
HabZIP14	158	17913.5	10.75	69.48	53.23	-0.97
HabZIP57	142	16402.06	9.92	68	83.73	-0.637
HabZIP23	273	30181.96	6.31	30	66.45	-0.662
HabZIP37	256	28882.28	9.22	52.56	58.95	-1.01
HabZIP15	280	31658.51	6.14	48.66	66.5	-0.912
HabZIP12	321	35540.78	9.24	52.8	64.98	-0.925
HabZIP66	298	33175.57	9.72	51.43	70.97	-0.788
HabZIP38	386	42554.89	9.75	40.66	64.35	-0.73
HabZIP28	422	45165.57	9.69	47.11	66.52	-0.582
HabZIP82	384	41681.01	5.54	58.03	63.2	-0.67
HabZIP36	390	41613.95	9.69	37.39	60.72	-0.628
HabZIP27	164	18405.75	9.37	61.16	60.73	-0.905
HabZIP56	160	17835.94	9.55	61.44	69.5	-1.054
HabZIP19	131	14692.96	10.25	45.4	84.12	-0.749
HabZIP67	444	47959.69	9.13	53.02	63.74	-0.713
HabZIP24	140	15853.14	9.45	43.02	86.21	-0.736
HabZIP13	222	25006.32	9.37	59.62	67.25	-0.815
HabZIP80	267	29294.3	5.94	62.93	46.44	-1.013
HabZIP39	382	41733.82	9.26	51.95	60	-0.73
HabZIP2	148	16695.84	6.12	47.86	85.07	-0.665
HabZIP54	348	36527	9.34	46.88	66.49	-0.678
HabZIP79	313	34245.63	5.11	54.36	59.81	-0.78
HabZIP3	183	21508.85	5.62	43.91	66.07	-0.972
HabZIP75	166	18610.85	6.3	52.28	64.04	-0.599
HabZIP61	277	30856.11	5.34	63.94	62.38	-0.829
HabZIP60	110	11941.69	10.88	46.36	78.73	-0.442
HabZIP68	347	37421.08	5.1	63.41	47.03	-0.948
HabZIP34	280	31957.38	5.63	57.47	61.68	-0.946
HabZIP53	147	16650.02	8.82	55.67	84.97	-0.614
HabZIP73	349	37239.83	5.59	56.79	40.66	-0.971
HabZIP7	138	16296.6	9.14	69.54	57.9	-0.962
HabZIP31	450	49250.36	7.2	48	78.42	-0.503
HabZIP63	443	48288.68	6.05	57.48	56.34	-0.832
HabZIP42	172	20102.31	5.96	60.08	70.76	-0.868
HabZIP72	325	35249.71	5.72	55.51	42.34	-1.007
HabZIP76	269	30209.34	5.64	69.36	64.91	-0.861

HabZIP11	175	20137.85	8.55	65.85	76.34	-0.605
HabZIP78	351	37376.84	5.87	57.98	38.43	-1.026
HabZIP9	353	39179.27	5.43	52.04	64.08	-0.666
HabZIP41	382	40875.69	5.98	59.2	43.51	-1.047
HabZIP64	159	17573.61	9.73	54.2	65.09	-1.066
HabZIP62	379	40738.31	6.48	57.16	53.64	-0.88
HabZIP43	193	22289.78	5.35	64.37	73.78	-0.78
HabZIP35	489	54328.64	9.5	45.7	79.75	-0.548
HabZIP29	413	45741.9	6.78	44.86	74.62	-0.67
HabZIP16	374	40299.22	5.64	65.25	47.78	-1.017
HabZIP46	508	56579.34	6.34	62.6	74.39	-0.627
HabZIP55	375	40188.65	6.74	55.27	56.83	-0.85
HabZIP58	210	24464.09	5.71	57.47	66.9	-0.894
HabZIP81	131	15095.16	6.91	57.89	80.31	-0.544
HabZIP8	164	19107.39	6.38	52.57	77.8	-0.765
HabZIP30	584	63658.61	7	59.17	54.18	-0.977
HabZIP52	296	32825.7	6.73	53.19	62.33	-0.765
HabZIP48	211	24592.26	5.75	60.38	59.62	-1.001
HabZIP74	325	36123.23	8.03	43.94	61.63	-0.786
HabZIP5	149	17372.86	10.09	59.82	90.2	-0.68
HabZIP70	169	19766.98	5.76	59.23	81.42	-0.851
HabZIP77	132	15033.52	5.67	72.2	57.65	-1.195
HabZIP6	152	17598.65	6.42	50.61	64.21	-0.971
HabZIP10	315	34663.08	5.75	48.36	59.78	-0.85
HabZIP21	512	56907.53	6.53	56.58	73.55	-0.578
HabZIP51	349	38470.02	6.61	48.27	65.21	-0.798
HabZIP26	328	36462.02	8.63	61.5	81.62	-0.53
HabZIP20	328	36392.94	7.9	59.46	83.38	-0.492
HabZIP71	340	37677.35	5.97	61.72	61.24	-0.908
HabZIP1	183	21331.21	10.14	65.71	71.91	-0.868
HabZIP25	167	19318.14	9.68	41.04	74.73	-0.712
HabZIP45	326	36480.08	8.63	60.54	81.23	-0.538
HabZIP69	424	46585.69	6.28	60.28	60.12	-0.847
HabZIP33	333	36436.1	5.84	50.5	69.79	-0.759
HabZIP4	175	19245.39	6.28	57.57	72.97	-0.507
HabZIP65	499	56214.97	6.75	60.01	73.51	-0.617
HabZIP32	338	37172.54	5.75	63.38	55.27	-0.894
HabZIP59	335	38627.19	6.21	57.77	64.63	-0.91
HabZIP44	178	20524.3	6.14	56.25	72.3	-0.357
HabZIP18	335	37343.03	5.96	50.39	62.45	-0.888

Table S2 Summary of clean reads in the axillary bud transcriptomes of Sunflower.

Sample name	Raw reads	Clean reads	UMI reads	Raw bases	Clean bases	Error rate(%)	Q20(%)	Q30(%)	GC content(%)	UMI2Clean (%)	Dedup2Mapped UMI(%)	Deduped ratio(%)
Dec 0 d 1	43445922	42830278	41707362	6.52G	6.42G	0.03	97.04	92.03	45.44	97.38	75.75	24.25
Dec 0 d 2	50078208	49272174	47934822	7.51G	7.39G	0.03	97.39	92.7	45.71	97.29	76.88	23.12
Dec 0 d 3	45716982	45123796	44077852	6.86G	6.77G	0.03	97.76	93.35	45.03	97.68	69.7	30.3
Dec 1 d 1	43840122	43243934	42171020	6.58G	6.49G	0.03	97.28	92.59	45.21	97.52	75.18	24.82
Dec 1 d 2	47869830	47267758	46168586	7.18G	7.09G	0.03	97.77	93.37	45.12	97.67	69.78	30.22
Dec 1 d 3	44088116	43556448	42528244	6.61G	6.53G	0.03	97.09	92.11	44.74	97.64	76.82	23.18
Dec 2 d 1	45769928	45159426	43931236	6.87G	6.77G	0.03	97.15	92.29	45.17	97.28	74.54	25.46
Dec 2 d 2	42009198	41468536	40389784	6.3G	6.22G	0.03	96.9	91.74	44.74	97.4	72.31	27.69
Dec 2 d 3	43012750	42227792	41178168	6.45G	6.33G	0.03	97.15	92.33	44.81	97.51	74.77	25.23
